# Macrolide-Resistant *Mycoplasma genitalium* in Southeastern Region of the Netherlands, 2014–2017

**DOI:** 10.3201/eid2511.190912

**Published:** 2019-11

**Authors:** Marta Adelantado, Ana Navascués, Xabier Beristain, Alberto Gil-Setas, Maria E. Portillo, Aitziber Aguinaga, Carmen Ezpeleta

**Affiliations:** Complejo Hospitalario de Navarra Department of Clinical Microbiology, Pamplona, Spain

**Keywords:** *Mycoplasma genitalium*, macrolide resistance, antimicrobial resistance, bacteria, sexually transmitted infections, the Netherlands

**To the Editor:** We read with interest the article by Martens et al., which analyzed the frequency of macrolide resistance–mediating mutations in *Mycoplasma genitalium* infections in the southeastern region of the Netherlands during 2014–2017 (*1*). The authors reported high rates of macrolide resistance in *M. genitalium* infections (281/827; 34.0%) and observed a decrease in the rate in 2017 (115/290; 39.7%) compared with 2016 (92/207; 44.4%), after an increase in the number of tests of cure performed.

Increasing rates of macrolide resistance in *M. genitalium* are a problem not only in the Netherlands but also worldwide: 77.4% in New Zealand (*2*) and 41% in the United Kingdom (*3*). Macrolide resistance in *M. genitalium* as a consequence of single-dose azithromycin treatment has been previously reported (*4*). European Academy of Dermatology and Venereology guidelines recommend azithromycin in an extended regimen (500 mg day 1, 250 mg days 2–5, orally) as a first-line treatment, followed by a test of cure 4–6 weeks after treatment (*5*).

In northern Spain, local protocols for the treatment of *M. genitalium* infections are based on the European guideline. We performed a prospective/retrospective study during August 2015–October 2018. We confirmed 173 cases of *M. genitalium* infection; mean patient age was 29.4 years, and 57.2% (99/173) were male. We found macrolide-resistant *M. genitalium* strains in 21.8% (27/124) patients, which is a lower rate than was found in the Netherlands. Most of the patients attending posttreatment follow-up showed wild-type *M. genitalium,* and only 10.9% (5/46) became resistant to azithromycin treatment, in contrast with 89.6% (60/67) reported by Martens et al. We suggest that the decrease in macrolide resistance resulted from the increased number of posttreatment follow-ups. Our data confirm this. We believe that giving local advice on the basis of extended azithromycin treatment and posttreatment follow-up can limit the spread of macrolide resistance.
